# Catheter navigation by intracardiac echocardiography enables zero-fluoroscopy linear lesion formation and bidirectional cavotricuspid isthmus block in patients with typical atrial flutter

**DOI:** 10.1186/s12947-023-00312-w

**Published:** 2023-08-03

**Authors:** Blerim Luani, Maksim Basho, Ammar Ismail, Thomas Rauwolf, Sven Kaese, Ndricim Tobli, Alexander Samol, Katharina Pankraz, Alexander Schmeisser, Marcus Wiemer, Rüdiger C. Braun-Dullaeus, Conrad Genz

**Affiliations:** 1 Department of Cardiology and Intensive Care Medicine, Johannes Wesling University Hospital Minden Ruhr-University Bochum, Hans-Nolte-Str. 1, Minden, 32429 Germany; 2 Department of Radiology, University Hospital Center Mother Teresa, Tirana, Albania; 3 Department of Internal Medicine, Division of Cardiology and Angiology, Magdeburg University, Magdeburg, Germany

**Keywords:** Catheter ablation, Cavotricuspid isthmus, Zero-fluoroscopy, Intracardiac echocardiography

## Abstract

**Introduction:**

One of the most helpful aspects of intracardiac echocardiography (ICE) implementation in electrophysiological studies (EPS) is the real-time visualisation of catheters and cardiac structures. In this prospective study, we investigated ICE-guided zero-fluoroscopy catheter navigation during radiofrequency (RF) ablation of the cavotricuspid isthmus (CTI) in patients with typical atrial flutter (AFL).

**Methods and results:**

Thirty consecutive patients (mean age 72.9 ± 11.4 years, 23 male) with ongoing (*n* = 23) or recent CTI-dependent AFL underwent an EPS, solely utilizing ICE for catheter navigation. Zero-fluoroscopy EPS could be successfully accomplished in all patients. Mean EPS duration was 41.4 ± 19.9 min, and mean ablation procedure duration was 20.8 ± 17.1 min. RF ablation was applied for 6.0 ± 3.1 min (50W, irrigated RF ablation). Echocardiographic parameters, such as CTI length, prominence of the Eustachian ridge (ER), and depth of the CTI pouch on the ablation plane, were assessed to analyse their correlation with EPS- or ablation procedure duration. The CTI pouch was shallower in patients with an ablation procedure duration above the median (4.8 ± 1.1 mm vs. 6.4 ± 0.9 mm, *p* = 0.04), suggesting a more lateral ablation plane in these patients, where the CTI musculature is stronger. CTI length or ER prominence above the respective median did not correlate with longer EPS duration.

**Conclusions:**

Zero-fluoroscopy CTI ablation guided solely by intracardiac echocardiography in patients with CTI-dependent AFL is feasible and safe. ICE visualisation may help to localise the optimal ablation plane, detect and correct poor tissue contact of the catheter tip, and recognise early potential complications during the ablation procedure.

**Graphical Abstract:**

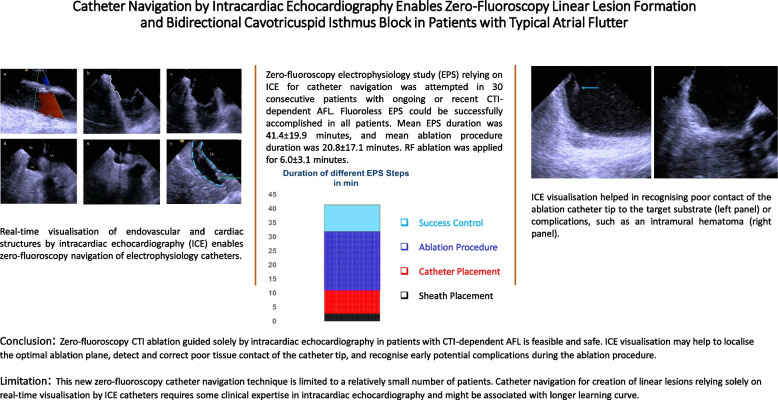

**Supplementary Information:**

The online version contains supplementary material available at 10.1186/s12947-023-00312-w.

## Introduction

Intracardiac echocardiography (ICE) has been implemented early in catheter ablation of cardiac arrhythmias [1]. Some of the advantages of using ICE in electrophysiology (EP) procedures are the ability to safely guide transseptal puncture for left atrial access, precise localisation of the ablation catheter tip in relation to the target substrate and important anatomical structures, early recognition of complications etc. [2, 3]. The real-time visualisation of anatomical landmarks and EP catheters via ICE facilitates ablation procedures both in patients with normal anatomy, as well as in those with untreated or corrected complex congenital heart diseases [4, 5].

Another advantage of ICE technology is the potential to reduce radiation exposure in EP procedures. A significant reduction in fluoroscopy time and dosage has been reported for cryo-balloon ablation in patients with atrial fibrillation when using ICE for balloon occlusion confirmation [6]. Furthermore, the combination of ICE with three-dimensional mapping systems enables zero- or near-zero-fluoroscopy ablation of left atrial or ventricular arrhythmias [7, 8]. In previous studies, we have demonstrated the feasibility and safety of ICE-guided focal cryothermal ablation of the slow pathway in patients with atrioventricular nodal re-entry tachycardia (AVNRT) [9, 10]. Compared to conventional fluoroscopic guidance, the ICE-guided real-time echocardiographic visualisation of the ablation catheter within the triangle of Koch may shorten the cryo-ablation procedure in AVNRT patients, as demonstrated in our previous work [10]. The advantage of reducing or avoiding fluoroscopy by ICE visualisation may be expanded in other EP procedures, especially in those with known arrhythmogenic substrate.

In this prospective study, we aimed to investigate the feasibility and safety of exclusively ICE-guided EP catheter navigation to create a linear lesion and bidirectional cavotricuspid isthmus block in patients with ongoing or documented typical atrial flutter.

## Methods

In this study, we enrolled consecutive patients with electrocardiographic (ECG) documentation of atrial flutter (AFL) suggesting cavotricuspid isthmus (CTI)-dependent AFL and indication for CTI ablation after giving their informed consent. Patients with transvenous leads, other implanted cardiac devices, previous cardiac surgery, or deformations of the spinal column were excluded from this study. Patients with insufficient oral or intravenous anticoagulation therapy in the last three weeks underwent a transoesophageal echocardiography (TEE) prior to the EP procedure to exclude intracardiac thrombi, except for those presenting sinus rhythm.

### Electrophysiological study

**Fig. 1:**
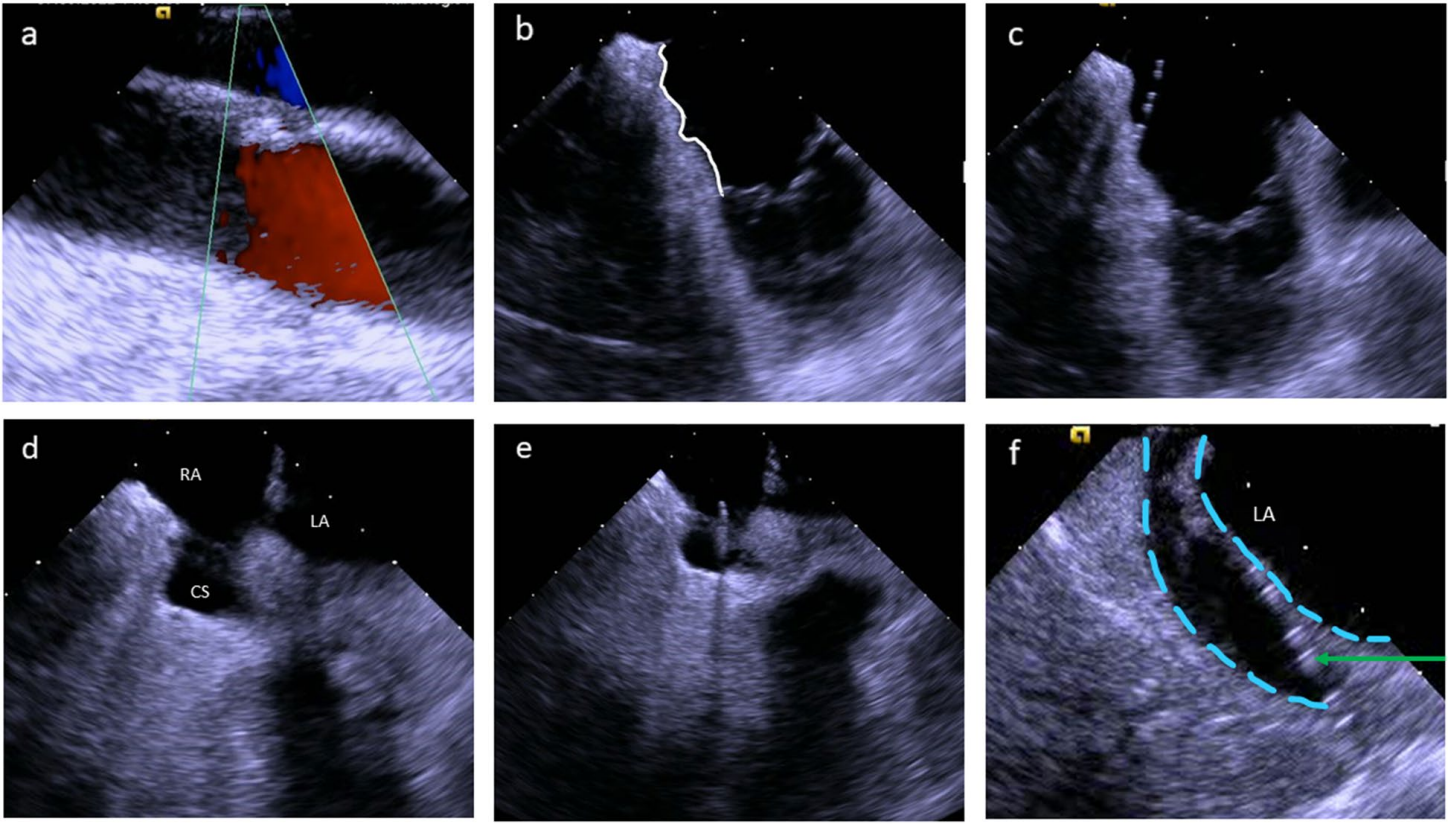
**a** View of the right iliac vein and artery; **b** typical CTI view (white line); **c** diagnostic EP catheter placed on the CTI for entrainment manoeuvre; **d** visualisation of the coronary sinus ostium (RA = right atrium, LA = left atrium, CS = coronary sinus); **e** decapolar diagnostic catheter manoeuvered into the coronary sinus ostium; **f** diagnostic catheter (green arrow) placed in the coronary sinus (blue dashed line)

Three commercial sheaths (two 8F and one 7F) were introduced into the right femoral vein after local anaesthesia using the Seldinger technique. Zero-fluoroscopy catheter navigation via ICE was attempted in all patients. Medical staff and patients did not wear x-ray protection equipment, but fluoroscopy and lead aprons were available as a bail-out navigation strategy. ICE was performed using a steerable catheter (8F, two-dimensional; AcuNav™, Biosense Webster, Inc.) coupled to the ACUSON SC2000 Prime echocardiographic system (Siemens AG, Munich, Germany). Two common EP catheters were employed to perform overdrive stimulation, differential pacing, and radiofrequency ablation of the CTI (6F decapolar steerable diagnostic catheter, Inquiry™ and 7F irrigated radiofrequency ablation catheter, Therapy™ Cool Flex™; both Abbott, Eschborn, Germany). We have described the navigation of the AcuNav™ and EP catheters in detail in our previous work [9]. The ICE-catheter was manoeuvered from the femoral vein to the right atrium by gentle advancement following the blood flow direction (as assessed by colour doppler) and continuous parallel adjustment of the ICE-catheter tip to the femoral, iliac, or inferior caval vein walls, (Fig. 1a). The real-time visualisation by ICE was used to safely guide the two other EP catheters to specific positions in the right atrium or coronary sinus.

If AFL was present at the beginning of the procedure, overdrive pacing at CTI locations were performed to confirm concealed entrainment and a post-pacing interval (PPI) equal or slightly longer than the AFL cycle length (CL), (Fig. 1b and c, Additional file 1). Thereafter, the Inquiry™ catheter was placed into the coronary sinus, (Fig. 1d–f).

**Fig. 2:**
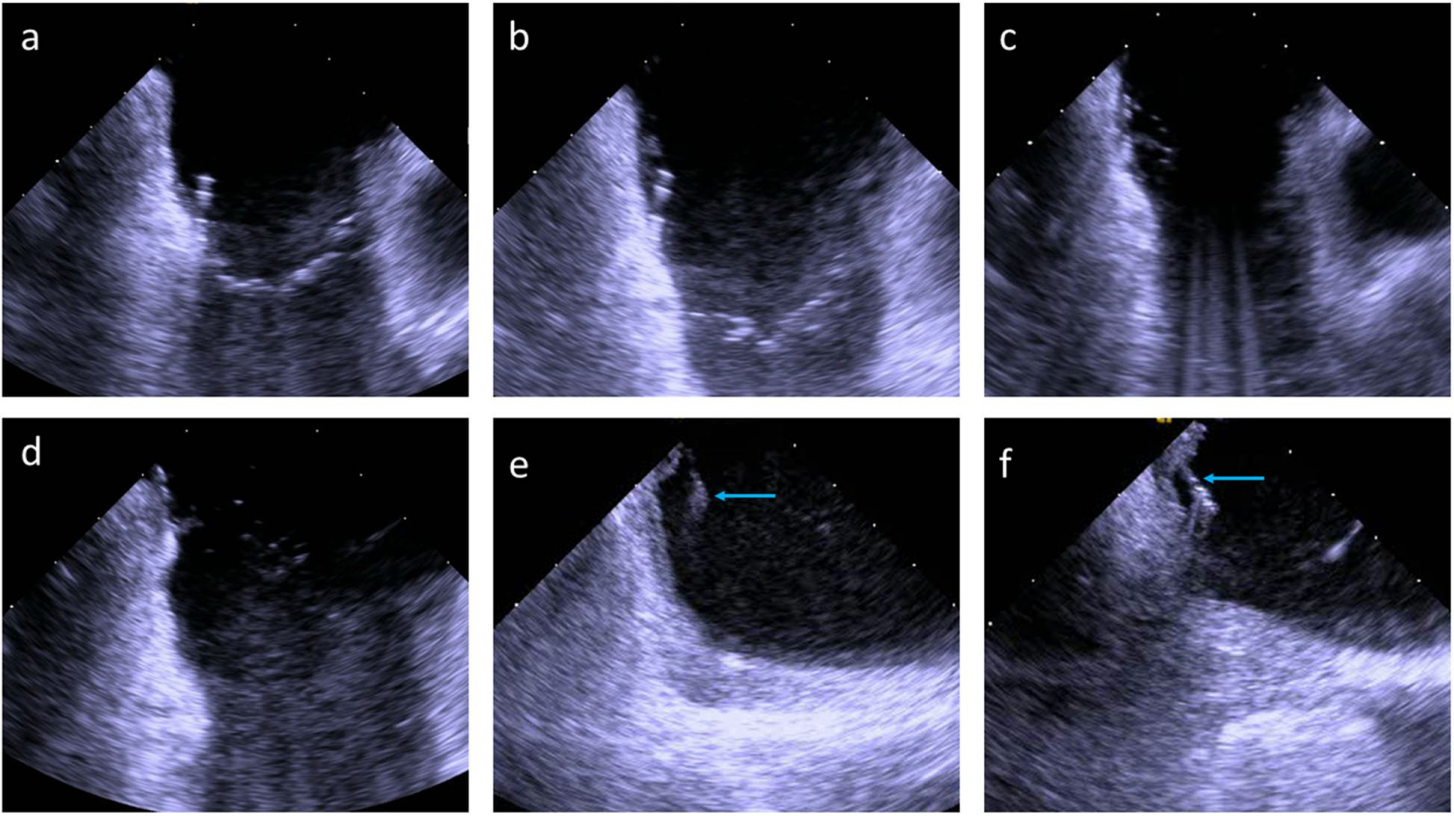
**a**–**d** Typical ablation planes with CTI on the left side. The ablation catheter is dragged from the tricuspid valve ring to the inferior caval vein, maintaining the catheter on the echocardiographic plane to achieve the best possible line; **e** insufficient contact of the ablation catheter tip (blue arrow) to the target tissue due to a prominent Eustachian ridge; **f** corrected tissue contact by inversion of the ablation catheter (blue arrow)

After confirmation of CTI- dependent AFL or in patients presenting sinus rhythm, CTI ablation was performed using 50 W, open irrigated radiofrequency current. The ablation catheter was dragged from the tricuspid anulus to the inferior caval vein at the lowest position on the standard ICE-plane, as seen in Fig. 2a–d and Additional files 2, 3, 4. The inversion manoeuvre was performed if poor contact of the catheter tip with the target substrate was noticed (Fig. 2e and f).

A straight ablation line was attempted by maintaining a stable echocardiographic plane and manoeuvring the ablation catheter on that plane. After completion of the ablation line, differential pacing was performed using the two mentioned EP catheters. In patients with no bidirectional CTI block after completion of the ablation line, further ablations guided by local potentials were performed. After successful ablation, pericardial effusion was excluded by ICE visualisation and a control transthoracic echocardiography (TTE) was performed before discharge from the hospital. A vascular ultrasound examination was performed if inspection or auscultation of the access side before discharge revealed a pathological result.

### Echocardiographic parameters

**Fig. 3:**
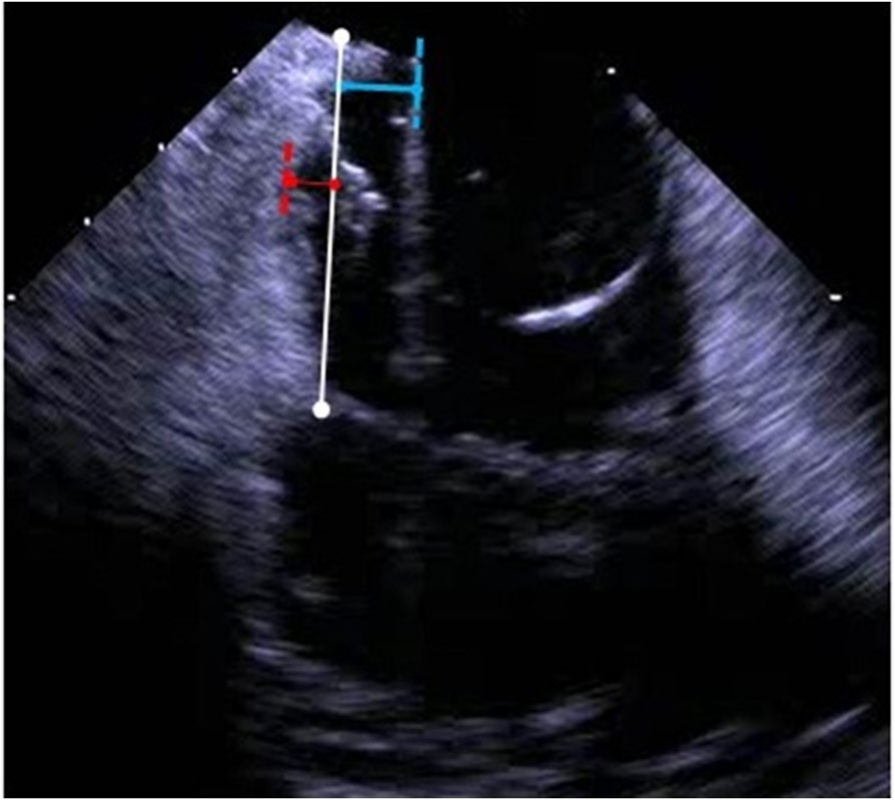
Red segment = depth of the CTI pouch; white segment = CTI length; blue segment = prominence of the Eustachian ridge

Different anatomic parameters were assessed by ICE to analyse their correlation with the duration of different steps or the entire EP procedure. CTI length was defined as the distance between the tricuspid valve ring and the inferior vena cava, while the depth of the CTI pouch was measured from the line connecting those two edges to the deepest CTI location. The prominence of the Eustachian ridge was measured from its highest location to the line connecting the two CTI edges, see Fig. 3 for details.

### Statistical analysis

Categorical parameters are presented as counts and percentages, whereas continuous variables are presented as mean values ± standard deviation. Spearman’s rank correlation was used to analyse the relationship between CTI length, depth of the CTI pouch, or prominence of the Eustachian ridge and the ablation or EP procedure duration. The t-test was used to calculate the level of significance; a *p*-value < 0.05 was considered statistically significant. Statistical analysis was performed using SPSS Version 27 (IBM, Armonk, NY, USA).

## Results

Zero-fluoroscopy CTI ablation was attempted in thirty consecutive patients (mean age 72.9 ± 11.4 years, 21 male) with ECG recordings suggesting ongoing (*n* = 23) or recent CTI-dependent AFL. Patients’ baseline characteristics are shown in Table 1.

**Table 1:** Patients’ baseline characteristics

Patients, *n*	30
Mean age, years	72.9 ± 11.4
Male, % (*n*)	76.7 (23)
BMI, kg/m^2^	28.9 ± 5.5
Coronary heart disease, % (n)	43.3 (13)
COLD, % (n)	16.7 (5)
Arterial hypertension % (n)	80 (24)
Diabetes mellitus % (n)	26.7 (8)
Hyperlipidemia % (n)	46.7 (14)
Smoking/Smoking history % (n)	13.3 (4)
History of atrial fibrillation % (n)	23.3 (7)
Echocardiographic parameters	
LVEF, %	50.0 ± 8.7
IVS, mm	11.6 ± 1.7
VCI, mm	15.1 ± 2.0
Estimated systolic PAP, mmHg	30.8 ± 4.9

**Fig. 4:**
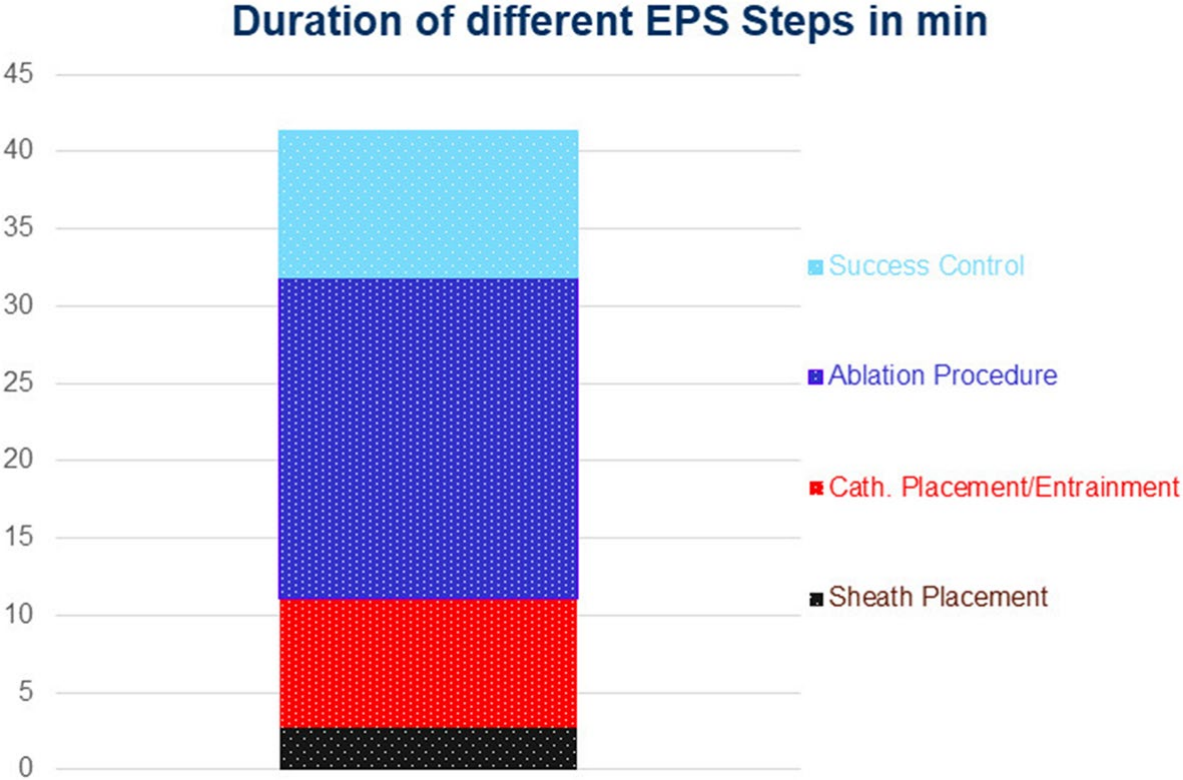
Duration of different EPS steps in detail

All EPSs could be successfully accomplished without the need for fluoroscopy, relying solely on ICE visualisation for catheter navigation. CTI-dependent AFL was confirmed by the entrainment manoeuvre in all patients with ongoing AFL. Mean EPS duration, defined as the time interval from the first venous puncture to removal of all sheaths, was 41.4 ± 19.9 min. Mean ablation procedure duration, defined as the time interval from the beginning of the first RF ablation to the end of the last one, was 20.8 ± 17.1 min. Figure 4 shows the duration of the different EPS steps in detail. RF ablation was applied for 6.0 ± 3.1 min (50 W, open irrigated RF ablation in all patients). After the last RF application, bidirectional CTI block was confirmed by differential pacing in all patients. Table 2 shows the electrophysiological parameters of all patients.

**Table 2:** Electrophysiological parameters

Patients, *n*	30
Ongoing CTI-dependent AFL	76.7 (23)
Cycle length of ongoing or documented AFL, ms	232.8 ± 18.3
PQ after CTI-Ablation, ms	198.6 ± 38.2
QRS before CTI-Ablation, ms	104.3 ± 25.1
QRS after CTI-Ablation, ms	102.8 ± 23.8
QT before CTI-Ablation, ms	364.9 ± 52.4
QT after CTI-Ablation, ms	390.6 ± 49.3
Heart rate before CTI-Ablation, bpm	110.8 ± 19.7
Heart rate after CTI-Ablation, bpm	62.8 ± 11.9
Differential pacing after CTI-ablation, intervals in ms	
CSOS – LRA	142.2 ± 13.2
CSOS – MRA	121.6 ± 12.8
LRA—CSOS	142.6 ± 13.3
RF-Applications (5–60 s), n	13.0 ± 6.2
Duration of RF-Application, min	6.0 ± 3.1

*CSOS* coronary sinus ostium, *LRA* low right atrium, *MRA* mid right atrium

In a series of 30 consecutive patients, who recently underwent fluoroscopy guided CTI ablation in our centre, mean EPS duration, mean ablation procedure duration and total RF ablation time were 34.7 ± 13.2, 20.1 ± 11.7 and 9.5 ± 5.0 min, respectively.

Mean CTI length and depth of the CTI pouch in the study group were 32.4 ± 7.2 mm and 5.5 ± 1.5 mm, respectively. The mean ER prominence was 5.0 ± 1.9 mm. The CTI pouch was shallower in patients with an ablation procedure duration above the median (4.8 ± 1.1 mm vs. 6.4 ± 0.9 mm, *p* = 0.04), while CTI length or ER prominence did not correlate with EPS duration. Table 3 shows the Spearman’s rank correlation coefficients and the level of significance between these echocardiographic parameters and the EPS or ablation procedure (ABL) duration.

**Table 3:** Correlation between echocardiographic parameters and EPS/ABL duration

	EPS	*p* value	ABL	*p* value
CTI-Length	-0.28	0.21	-0.27	0.24
CTI-Pouch	-0.44	**0.04**	-0.43	**0.04**
ER-Prominence	-0.19	0.40	-0.05	0.83

**Fig. 5:**
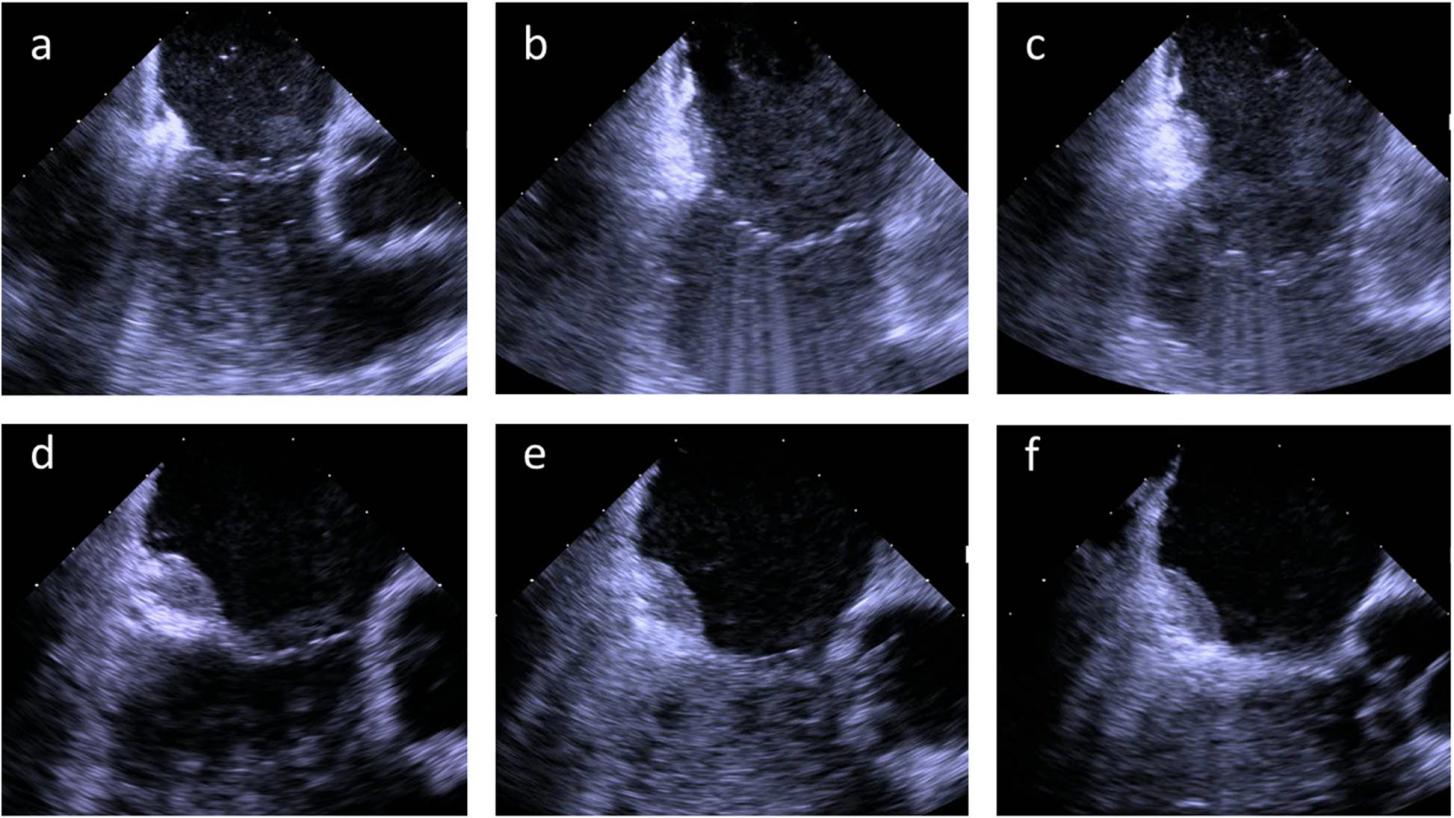
**a** RF ablation at the tricuspid valve (TV)–CTI junction; **b**, **c** ablation catheter dragged back to mid CTI, development of intramural hematoma at TV-CTI junction is noticed; **d** maximum size of intramural hematoma; **e**, **f** stable size of intramural hematoma throughout the rest of the procedure

No pericardial effusion, vascular complications, or electrical disturbances were observed in the study population. One patient developed an intramural hematoma during ablation, but it remained asymptomatic and constant throughout the procedure (see Fig. 5 and Additional file 5 for details). At the end of the procedure or the day thereafter, the intramural hematoma could not be detected by TTE.

No other major or minor complications were observed in this study. All patients could be discharged from the hospital the day after the procedure after excluding procedure related pathological results in the control ECG or transthoracic echocardiography.

## Discussion

In this prospective series of patients with CTI-dependent AFL, we demonstrated the feasibility of ICE-guided catheter navigation to achieve a successful zero-fluoroscopy ablation procedure within a reasonable time interval and with no complications related to the navigation technique.

ICE is available in most modern high- or medium-volume EP centres, and the advantages of an excellent real-time visualisation of the ablation catheter, target substrate, or anatomical landmarks by ICE has been previously reported for different ablation procedures [7, 11, 12]. Navigation of the ablation catheters or ICE catheter itself in those studies has been mostly guided by fluoroscopy or three-dimensional electro-anatomical mapping (3D EAM). Navigation of ICE or EP catheters relying entirely on intravascular or intracardiac echocardiographic imaging requires good anatomical knowledge and expertise in ICE-catheter control. As operators in this study had previously experienced ICE-guided zero-fluoroscopy ablation of the slow pathway in AVNRT patients [9, 10], they did not face any difficulties in manoeuvring the catheters to the specific regions in the right atrium or coronary sinus; however, the simultaneous usage of an additional catheter forces the operator to switch back-and-forth between the ablation and ICE catheter or requires a second operator to maintain a stable two-dimensional echocardiographic plane. This hurdle could be overcome by the implementation of three-dimensional ICE catheters with broad echocardiographic volumes or ICE catheter robotic control systems with automated catheter tip repositioning, which has been successfully used in heart phantoms and animal experiments [13].

Due to its high reputation in early recognition and reduction of potential complications during ablation procedures, ICE has been increasingly used in EP laboratories for the last few years. Not only can the usage of ICE significantly reduce the risk of cardiac perforation, especially during transseptal puncture [14], it can immediately reveal intracardiac thrombus formation during EP procedures as well [15], enabling a quick initiation of specific therapeutic measures. In our study, we did not face any severe complications, such as cardiac perforation, development of pericardial effusion, or intracardiac thrombus formation, as CTI RF ablation is a relatively short and commonly safe procedure. However, we documented the asymptomatic development of an intramural hematoma after an unobtrusive RF ablation on the tricuspid valve-CTI junction in one case, a complication that might happen more often than we assume and remain unrecognised as its diagnosis without ICE control could be difficult. It certainly needs to be proved in large studies if the ability of real-time visualisation of potential complications outweighs the disadvantages of using an additional vascular access side and an 8F catheter (ICE catheter).

Besides the efficacy and safety, overall ablation time, ablation procedure and EPS duration are important aspects while introducing a novel, though simple, zero-fluoroscopy ICE-guided navigation technique for CTI ablation. Technical development, modern ablation catheters, and increasing experience in electrophysiology have led to shorter ablation procedures or EPS duration in patients with CTI-dependent AFL. In most recent studies, the ablation time in patients who underwent RF CTI ablation with common open irrigated RF catheters ranged from 10 to 15 min [16–18]. In a large representative series of 1,051 patients, Kakehashi et al. reported a total radiofrequency time of 10.3 ± 6.6 min to achieve bidirectional CTI block [16]. Katritsis and Bacillieri report similar RF delivery time across the CTI in smaller series of patients with CTI-dependent AFL (12.2 and 10.7 min respectively) [17, 18]. Using contact force control and ablation- or lesion-size index (AI, LSI), as well as novel ablation strategies and catheters, such as high/very-high-power short-duration RF ablation or the diamond temp ablation catheter, seems to significantly shorten the RF delivery time in these patients, but their usage is inevitably related to higher costs [19–21]. In our series, the total radiofrequency time was 6.0 ± 3.1 min with a mean RF application number of 13.0 ± 6.2 and, therefore, markedly shorter as in the mentioned studies and our series of patients with fluoroscopy guided CTI ablation. This might be explained by the continuous ICE-guided visualisation of the anatomy and avoidance of unnecessary and ineffective ablations in sites with poor tissue contact, as illustrated in Fig. 2e. Whether the above-mentioned novel ablation techniques or usage of contact force catheters could further reduce the total radiofrequency time in the setting of ICE-guided catheter navigation in patients with CTI-dependent AFL should be investigated in further studies. The mean ablation procedure duration and mean EPS duration in our series, with 20.8 ± 17.1 and 41.4 ± 19.9 min, respectively, lie well within the range of the same parameters in the mentioned studies as well. Due to the more challenging catheter placement when relying solely on ICE visualisation, mean EPS duration in the ICE-guided group was slightly longer than the one in the mentioned series of fluoroscopy guided CTI ablation in our centre (41.4 ± 19.9 vs. 34.7 ± 13.2, *p* < 0.05).

As with most cardiological procedures, mean fluoroscopy time during CTI ablation depends on many factors, such as the investigators or centre’s experience, usage of 3D EAM etc. In the study of Golian et al. mean fluoroscopy time and dose ranged from 34 ± 12 to 36 ± 21 min and 728 ± 1240 to 816 ± 1011 mGycm^2^, whereas in our series of fluoroscopy guided CTI ablation mean fluoroscopy time and dose were 9.4 ± 6.6 min and 95.3 ± 73.4 cGycm^2^, respectively. In the ICE-guided group fluoroscopy could be entirely avoided by the new zero-fluoroscopy catheter navigation technique.

In our study, the CTI pouch was shallower in patients with an ablation procedure duration above the median, suggesting a more lateral ablation plane in these patients where the CTI musculature is stronger. It must be mentioned that each RF application was performed at the discretion of the operator, evaluating the local signals derived by the ablation catheter in real time. Using AI- or LSI-guided ablation may have a different impact on the ablation procedure or EPS duration.

## Limitations

This new zero-fluoroscopy catheter navigation technique is limited to a relatively small number of patients with typical atrial flutter. Catheter navigation for creation of linear lesions relying solely on real-time visualisation by ICE catheters requires some clinical expertise in intracardiac echocardiography and its broad implementation might be associated with longer procedures or the need for additional imaging technologies, such as fluoroscopy or three-dimensional mapping.

## Conclusions

Zero-fluoroscopy CTI ablation guided solely by intracardiac echocardiography in patients with CTI-dependent AFL is feasible and safe. ICE visualisation may help to localise the optimal ablation plane, detect and correct poor tissue contact of the catheter tip, and recognise early potential complications during the ablation procedure.

### Supplementary Information

**Additional file 1.** 

**Additional file 2.** 

**Additional file 3.** 

**Additional file 4.** 

**Additional file 5.**

## Data Availability

The data underlying this article will be shared on reasonable request to the corresponding author.

## References

[1] Chu E, Kalman JM, Kwasman MA, Jue JC, Fitzgerald PJ, Epstein LM, Schiller NB, Yock PG, Lesh MD (1994). Intracardiac echocardiography during radiofrequency catheter ablation of cardiac arrhythmias in humans. J Am Coll Cardiol.

[2] Szili-Torok T, McFadden EP, Jordaens LJ, Roelandt JR (2004). Visualization of elusive structures using intracardiac echocardiography: insights from electrophysiology. Cardiovasc Ultrasound.

[3] Nagy LT, Jenei C, Papp TB, Urbancsek R, Kolozsvari R, Racz A, Raduly AP, Veisz R, Csanadi Z (2023). Three-dimensional transesophageal echocardiographic evaluation of pulmonary vein anatomy prior to cryoablation: validation with cardiac CT scan. Cardiovasc Ultrasound.

[4] Peichl P, Kautzner J, Gebauer R (2009). Ablation of atrial tachycardias after correction of complex congenital heart diseases: utility of intracardiac echocardiography. Europace.

[5] Lin J, Cai Y, Meng X, Liu S, Wang F, Liu L, Zhu Z, Liu M, Ding L, Wu W, Wang H, Yao Y (2023). Left atrial reservoir strain measurements derived from intracardiac echocardiography in patients with atrial fibrillation: comparison with transthoracic echocardiography. Cardiovasc Ultrasound.

[6] Rubesch-Kütemeyer V, Fischbach T, Guckel D, Körber B, Horstkotte D, Gutleben KJ, Nölker G (2020). Long-term development of radiation exposure, fluoroscopy time and contrast media use in daily routine in cryoballoon ablations after implementation of intracardiac echocardiography and other radioprotective measures: experiences from a large single-centre cohort. J Interv Card Electrophysiol.

[7] Enriquez A, Saenz LC, Rosso R, Silvestry FE, Callans D, Marchlinski FE, Garcia F (2018). Use of intracardiac echocardiography in interventional cardiology: working with the anatomy rather than fighting it. Circulation.

[8] Khaykin Y, Skanes A, Whaley B, Hill C, Beardsall M, Seabrook C, Wulffhart Z, Oosthuizen R, Gula L, Verma A (2008). Real-time integration of 2D intracardiac echocardiography and 3D electroanatomical mapping to guide ventricular tachycardia ablation. Heart Rhythm.

[9] Luani B, Zrenner B, Basho M, Genz C, Rauwolf T, Tanev I, Schmeisser A, Braun-Dullaeus RC (2018). Zero-fluoroscopy cryothermal ablation of atrioventricular nodal re-entry tachycardia guided by endovascular and endocardial catheter visualization using intracardiac echocardiography (Ice&ICE Trial). J Cardiovasc Electrophysiol.

[10] Luani B, Rauwolf T, Genz C, Schmeißer A, Wiemer M, Braun-Dullaeus RC (2019). Intracardiac echocardiography versus fluoroscopy for endovascular and endocardial catheter navigation during cryo-ablation of the slow pathway in AVNRT patients. Cardiovasc Ultrasound.

[11] Kaplan RM, Narang A, Gay H, Gao X, Gibreal M, Arora R, Chicos A, Kim S, Passman R, Patil K, Pfenniger A, Verma N, Lin A, Knight BP (2021). Use of a novel 4D intracardiac echocardiography catheter to guide interventional electrophysiology procedures. J Cardiovasc Electrophysiol.

[12] Gianni C, Sanchez JE, Della Rocca DG, Al-Ahmad A, Horton RP, Di Biase L, Natale A (2021). Intracardiac echocardiography to guide catheter ablation of atrial fibrillation. Card Electrophysiol Clin.

[13] Kim YH, Collins J, Li Z, Chinnadurai P, Kapoor A, Lin CH, Mansi T (2022). Automated catheter tip repositioning for intra-cardiac echocardiography. Int J Comput Assist Radiol Surg.

[14] Friedman DJ, Pokorney SD, Ghanem A, Marcello S, Kalsekar I, Yadalam S, Akar JG, Freeman JV, Goldstein L, Khanna R, Piccini JP (2020). Predictors of cardiac perforation with catheter ablation of atrial fibrillation. JACC Clin Electrophysiol.

[15] Tonegawa-Kuji R, Yamagata K, Suzuki S, Miyazaki Y, Ueda N, Kusano K (2021). Prompt recognition and successful aspiration of a left atrial thrombus under intracardiac echocardiography guidance during radiofrequency catheter ablation for atrial tachycardia. Europace.

[16] Kakehashi S, Miyazaki S, Hasegawa K, Nodera M, Mukai M, Aoyama D, Nagao M, Sekihara T, Eguchi T, Yamaguchi J, Shiomi Y, Tama N, Ikeda H, Ishida K, Uzui H, Tada H (2022). Safety and durability of cavo-tricuspid isthmus linear ablation in the current era: Single-center 9-year experience from 1078 procedures. J Cardiovasc Electrophysiol.

[17] Baccillieri MS, Rizzo S, De Gaspari M, Paradiso B, Thiene G, Verlato R, Basso C (2019). Anatomy of the cavotricuspid isthmus for radiofrequency ablation in typical atrial flutter. Heart Rhythm.

[18] Katritsis DG, Chokesuwattanaskul R, Zografos T, Jame S, Paxinos G, Morady F (2022). A simplified differential pacing technique for the evaluation of bidirectional cavo-tricuspid isthmus block during ablation of typical atrial flutter. J Interv Card Electrophysiol.

[19] Golian M, Ramirez FD, Alqarawi W, Hansom SP, Nery PB, Redpath CJ, Nair GM, Shaw GC, Davis DR, Birnie DH, Sadek MM (2020). High-power short-duration radiofrequency ablation of typical atrial flutter. Heart Rhythm O2.

[20] Viola G, Stabile G, Bandino S, Rossi L, Marrazzo N, Pecora D, Bottoni N, Solimene F, Schillaci V, Scaglione M, Ocello S, Baiocchi C, Santoro A, Donzelli S, De Ruvo E, Lavalle C, Sanchez-Gomez JM, Pastor JFA, Grandio PC, Ferraris F, Castro A, Rebellato L, Marchese P, Adao L, Primo J, Barra S, Casu G (2021). Safety, efficacy, and reproducibility of cavotricuspid isthmus ablation guided by the ablation index: acute results of the FLAI study. Europace.

[21] Ramak R, Lipartiti F, Mojica J, Monaco C, Bisignani A, Eltsov I, Sorgente A, Capulzini L, Paparella G, Deruyter B, Iacopino S, Motoc AI, Luchian ML, Osorio TG, Overeinder I, Bala G, Almorad A, Ströker E, Sieira J, Jordaens L, Brugada P, de Asmundis C, Chierchia GB (2022). Comparison between the novel diamond temp and the classical 8-mm tip ablation catheters in the setting of typical atrial flutter. J Interv Card Electrophysiol.

