# Supplementary figures and images for: Catheter navigation by intracardiac echocardiography enables zero-fluoroscopy linear lesion formation and bidirectional cavotricuspid isthmus block in patients with typical atrial flutter
Source: Cardiovasc Ultrasound. 2023 Aug 3;21:13. doi: 10.1186/s12947-023-00312-w (PMC10398930; doi:10.1186/s12947-023-00312-w)

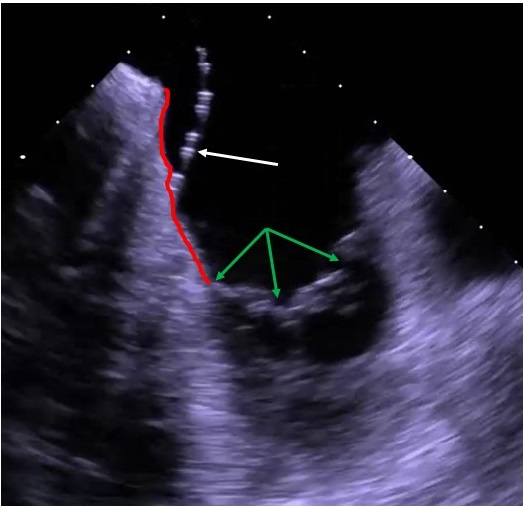

Supplement: Supplementary file 1 — Additional file 1. [file 12947_2023_312_MOESM1_ESM.zip › Clip 1 Capture.jpg]

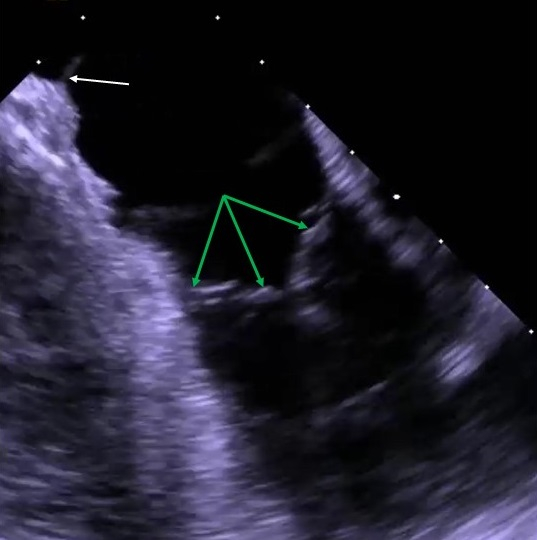

Supplement: Supplementary file 2 — Additional file 2. [file 12947_2023_312_MOESM2_ESM.zip › Clip 2 Capture.jpg]

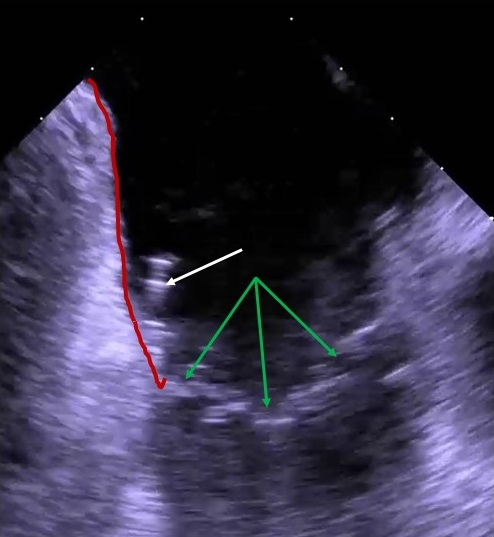

Supplement: Supplementary file 3 — Additional file 3. [file 12947_2023_312_MOESM3_ESM.zip › Clip 3 Capture.jpg]

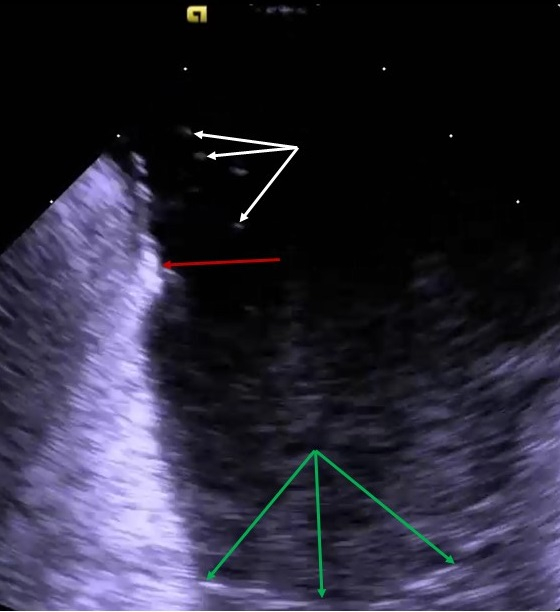

Supplement: Supplementary file 4 — Additional file 4. [file 12947_2023_312_MOESM4_ESM.zip › Clip 4 Capture.jpg]

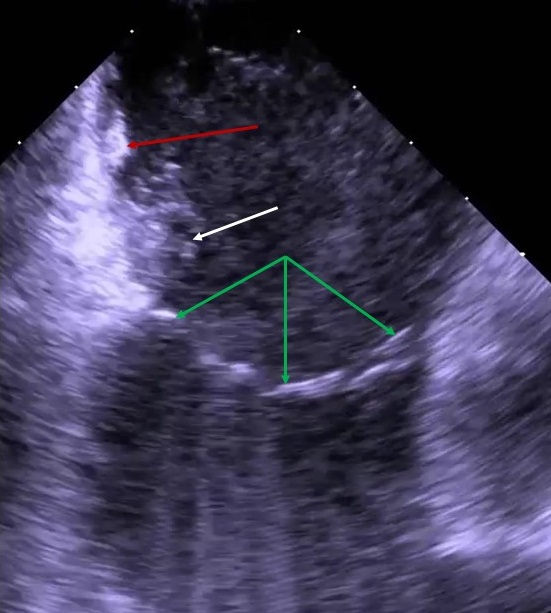

Supplement: Supplementary file 5 — Additional file 5. [file 12947_2023_312_MOESM5_ESM.zip › Clip 5 Capture.jpg]
